# Supplementary material for: Ultrasonographic and pathological correlation of asymmetric retroareolar density on mammogram
Source: PLoS One. 2024 Dec 5;19(12):e0301180. doi: 10.1371/journal.pone.0301180 (PMC11620652; doi:10.1371/journal.pone.0301180)
Supplement: Attachment — Submitted filename: response to reviewers comment feb.docx [file pone.0301180.s002.docx]

Reviewers' comments:

Reviewer's Responses to Questions

**Comments to the Author**

1. If the authors have adequately addressed your comments raised in a previous round of review and you feel that this manuscript is now acceptable for publication, you may indicate that here to bypass the “Comments to the Author” section, enter your conflict of interest statement in the “Confidential to Editor” section, and submit your "Accept" recommendation.

Reviewer #1: All comments have been addressed

Reviewer #2: All comments have been addressed

2. Is the manuscript technically sound, and do the data support the conclusions?

The manuscript must describe a technically sound piece of scientific research with data that supports the conclusions. Experiments must have been conducted rigorously, with appropriate controls, replication, and sample sizes. The conclusions must be drawn appropriately based on the data presented.

Reviewer #1: Yes

Reviewer #2: Yes

3. Has the statistical analysis been performed appropriately and rigorously?

Reviewer #1: Yes

Reviewer #2: N/A

4. Have the authors made all data underlying the findings in their manuscript fully available?

The [PLOS Data policy](http://www.plosone.org/static/policies.action#sharing) requires authors to make all data underlying the findings described in their manuscript fully available without restriction, with rare exception (please refer to the Data Availability Statement in the manuscript PDF file). The data should be provided as part of the manuscript or its supporting information, or deposited to a public repository. For example, in addition to summary statistics, the data points behind means, medians and variance measures should be available. If there are restrictions on publicly sharing data—e.g. participant privacy or use of data from a third party—those must be specified.

Reviewer #1: Yes

Reviewer #2: Yes

5. Is the manuscript presented in an intelligible fashion and written in standard English?

PLOS ONE does not copyedit accepted manuscripts, so the language in submitted articles must be clear, correct, and unambiguous. Any typographical or grammatical errors should be corrected at revision, so please note any specific errors here.

Reviewer #1: Yes

Reviewer #2: Yes

6. Review Comments to the Author

Please use the space provided to explain your answers to the questions above. You may also include additional comments for the author, including concerns about dual publication, research ethics, or publication ethics. (Please upload your review as an attachment if it exceeds 20,000 characters)

Reviewer #1: in page 7: "Using Fisher’s exact test, the odds
126 of grade 1 asymmetry to be a malignant lesion was significantly low (p= 0.00)". It would be appropriate to write the p value as <0.001

Author’s Response: Thank you for the feedback. Change has been made to address the comment (line 119).

Reviewer #2: Thank you for addressing the comments. I think including the raw table of statistical analysis is not appropriate for a publication. The final p-value should be put in the data table and the manuscript should just state which test was used without including the raw statistical program tabulation of the analysis. The software, and its version, used for statistical analysis should also be stated in the methodology
I think more emphasis should be given on the 21 patients who were diagnosed with malignancy, especially the four patients who the author described as having grade 2 lesions on radiology. Their presentation, work up and surgery and further treatment should be elaborated on to add value to the paper.

Authors’ response: The table has been removed and data has been presented in the text (line 119). The test performed and software used has been added (line 94) Additional information on clinical course of the patients with malignancy have been included (Line 121-128)

7. PLOS authors have the option to publish the peer review history of their article ([what does this mean?](https://journals.plos.org/plosone/s/editorial-and-peer-review-process#loc-peer-review-history)). If published, this will include your full peer review and any attached files.


If you choose “no”, your identity will remain anonymous but your review may still be made public.


**Do you want your identity to be public for this peer review?** For information about this choice, including consent withdrawal, please see our [Privacy Policy](https://www.plos.org/privacy-policy).

Reviewer #1: No

Reviewer #2: No
